# Optimizing the Rheological and Thermomechanical Response of Acrylonitrile Butadiene Styrene/Silicon Nitride Nanocomposites in Material Extrusion Additive Manufacturing

**DOI:** 10.3390/nano13101588

**Published:** 2023-05-09

**Authors:** Markos Petousis, Nikolaos Michailidis, Vassilis M. Papadakis, Apostolos Korlos, Nikolaos Mountakis, Apostolos Argyros, Evgenia Dimitriou, Chrysa Charou, Amalia Moutsopoulou, Nectarios Vidakis

**Affiliations:** 1 Department of Mechanical Engineering, Hellenic Mediterranean University, 71410 Heraklion, Greece; markospetousis@hmu.gr (M.P.); mountakis@hmu.gr (N.M.); charou@hmu.gr (C.C.); amalia@hmu.gr (A.M.); 2 Physical Metallurgy Laboratory, Mechanical Engineering Department, School of Engineering, Aristotle University of Thessaloniki, 54124 Thessaloniki, Greece; nmichail@auth.gr (N.M.); aargyros@auth.gr (A.A.); evgeniaod@auth.gr (E.D.); 3 Centre for Research & Development of Advanced Materials (CERDAM), Center for Interdisciplinary Research and Innovation, Balkan Centre, Building B’, 10th km Thessaloniki-Thermi Road, 57001 Thessaloniki, Greece; 4 Institute of Electronic Structure and Laser of the Foundation for Research and Technology-Hellas (IESL-FORTH), N. Plastira 100m, 70013 Heraklion, Greece; billyp@iesl.forth.gr; 5 Department of Industrial Design and Production Engineering, University of West Attica, 12243 Athens, Greece; 6 Department of Industrial Engineering and Management, International Hellenic University, 14th km Thessaloniki-N. Moudania, 57001 Thermi, Greece; apkorlos@ihu.gr

**Keywords:** additive manufacturing (AM), material extrusion (MEX), fused filament fabrication (FFF), 3D printing, acrylonitrile butadiene styrene (ABS), silicon nitride (Si_3_N_4_), mechanical properties

## Abstract

The current research aimed to examine the thermomechanical properties of new nanocomposites in additive manufacturing (AM). Material extrusion (MEX) 3D printing was utilized to evolve acrylonitrile butadiene styrene (ABS) nanocomposites with silicon nitride nano-inclusions. Regarding the mechanical and thermal response, the fabricated 3D-printed samples were subjected to a course of standard tests, in view to evaluate the influence of the Si_3_N_4_ nanofiller content in the polymer matrix. The morphology and fractography of the fabricated filaments and samples were examined using scanning electron microscopy and atomic force microscopy. Moreover, Raman and energy dispersive spectroscopy tests were accomplished to evaluate the composition of the matrix polymer and nanomaterials. Silicon nitride nanoparticles were proved to induce a significant mechanical reinforcement in comparison with the polymer matrix without any additives or fillers. The optimal mechanical response was depicted to the grade ABS/Si_3_N_4_ 4 wt. %. An impressive increase in flexural strength (30.3%) and flexural toughness (47.2%) was found. The results validate that these novel ABS nanocomposites with improved mechanical properties can be promising materials.

## 1. Introduction

Additive manufacturing, utilizing layer-by-layer deposition technology, is a groundbreaking and distinguished technique that enables the creation of three-dimensional objects with complex structures and diverse properties [1]. At present, diverse methods, including powder bed fusion (PBF), material extrusion (MEX), and vat photopolymerization (VVP), have been developed to manufacture stereoscopic objects with intricate geometry [2,3,4,5]. Additive manufacturing offers considerable advantages over removal or subtractive manufacturing techniques due to minimal waste material produced, as exemplified by the limited use of supports and brims [1]. To date, additive manufacturing has proven effective in meeting the demands for prototypes, customized products, and mass production processes, especially using fused filament fabrication (FFF) MEX technology [6]. The escalating demand for products with enhanced complexity and multi-functionality across a variety of industries [7], such as engineering [8], electronics [9], aerospace [10], and the medical field [11,12,13,14], has led to the acceptance and integration of this technology [15].

Convenient processing of polymers and reinforced polymers can be accomplished using the FFF technology [16]. This is due to its straightforward processing procedure, which involves depositing an extruded thermoplastic filament through a nozzle of accurate diameter in individual layers to construct a 3D object [17]. FFF is among the most frequently employed additive manufacturing processes [18]. The FFF technique can handle a broad range of thermoplastic filaments [19], including acrylonitrile butadiene styrene (ABS) and Polylactic Acid (PLA) [20,21]. Numerous studies have demonstrated that FFF manufacturing and structural factors, including printing speed, layer thickness, and temperature (at the bed, nozzle, and chamber) among others [19], are critical as they can meaningfully influence the mechanical behavior of the 3D-printed specimen and directly impact the product’s efficiency [15]. The relationship between these complex and diverse parameters and their properties has been extensively researched and analyzed [22,23]. It should be emphasized that FFF 3D printing presents significant challenges such as rough surface finish and porosity [24,25]. Research is aiming to reduce the effects of these parameters on the quality of the 3D-printed parts by optimizing the 3D printing settings used [26]. At the same time, MEX 3D printing is combined with other manufacturing techniques in a hybrid additive manufacturing system to further increase the quality of the built parts [27] and expand the potential fields of application [28].

The majority of studies examining the characteristics of polymers 3D printed using FFF technology have focused on acrylonitrile butadiene styrene materials due to their low melting point of approximately 200 °C and ease of use in printing [29]. ABS possesses advantageous traits such as being lightweight, chemically inert, environmentally friendly, and capable of being reused, making it well-suited for printing purposes [29,30,31]. ABS also displays commendable mechanical attributes, encompassing strength and toughness, as well as remarkable resistance to impacts and scratching [15,32]. It is the second most popular polymer in FFF 3D printing [33] and, as expected, it has been thoroughly investigated for its performance in the process. The sustainability of the polymer after multiple recycling courses has been investigated, proving it can withstand six thermomechanical repetitions [33]. Toward the evaluation of the polymers’ sustainability, the energy consumption for the manufacturing of parts with the FFF process has been evaluated by optimizing at the same time the mechanical performance of the parts [34,35]. Its mechanical properties have been studied under different strain rates [36,37] in tensile tests, in creep and fatigue loadings [38], in flexural tests using statistical modeling tools [39], and under impact loading [40], among others. Additionally, to enhance the mechanical properties of the polymer and induce multi-functional properties in some cases, composites have been developed employing ABS as the matrix material and mainly nano-scale (or in some cases micro-scale) additives, such as ceramics (titanium nitride) [15], carbon-based fillers (graphene [41], single and multi-wall carbon nanotubes [42]), metals (such as copper [43,44]) and metal oxides (such as antimony and titanium oxide [45,46]), and zinc oxide [46,47].

Gu [48] highlighted that materials play a crucial role in additive manufacturing (AM) technology, and the creation of new matrices could broaden the scope of three-dimensional printing (3DP) and offer new prospects for the advancement of additive manufacturing (AM) [49]. As AM continues to advance in various fields, there is a growing demand for suitable materials. Researchers are continuously exploring new materials, including nanomaterials, smart materials, and biomaterials [50,51,52,53]. Numerous research studies have indicated that incorporating additives with outstanding properties during MEX 3D printing can facilitate the production of polymer composites with remarkable characteristics [15]. Thus far, a diverse range of nanofillers has been employed to blend with conventional thermoplastics, resulting in filaments with distinct physical, thermal, electrical, and mechanical attributes [54]. In an earlier study, this same research team found that incorporating a ceramic filler, such as TiN nanoparticles into the ABS matrix resulted in a considerable improvement in mechanical performance, particularly in flexural properties when compared to pure ABS [15].

Numerous studies have established that the addition of nitride nanoparticles is a highly effective method for enhancing the overall characteristics and behavior of polymer nanocomposites in terms of thermomechanical properties [55,56,57]. These particles are excellent reinforcement materials [16]. The combination of polymer and ceramic in composites is anticipated to bring together the advantages of both materials [58]. Earlier research has stated that silicon nitride (Si_3_N_4_) ceramics demonstrate outstanding characteristics, including but not limited to high durability, potency, ability to withstand cracks, and sudden temperature changes [55,59,60]. As a result, they are widely utilized in a variety of applications such as spark plugs [61,62], cutting tools, hard coatings [63] and bearings [64], and heat engine components [65,66,67]. Apart from their exceptional mechanical properties that arise from the presence of strong covalent chemical bonds [55], silicon nitride (Si_3_N_4_) ceramics have become increasingly popular in the field of biomedicine owing to their inherent bio inertness and favorable biocompatibility. Moreover, studies have reported that Si_3_N_4_ ceramics exhibit bacteriostatic properties, indicating their potential for use in various biomedical applications [59,60].

In a recent study by Wang et al., silicon nitride (Si_3_N_4_) ceramics with porous structures were produced using gel casting and solid-state sintering techniques. Following this, the process of infusing polymers into the ceramic structure was performed, and the resulting composites were achieved by polymer curing after the infiltration process. The resulting composites, called PISNCs, exhibited a high level of resistance to bending, and a moderate level of hardness and elasticity. For instance, PISNCs obtained from SN60 achieved a flexural strength as high as 385.3 MPa, with the polymer constituting nearly 43% of the volume [59].

In their research, Kumar and Reddy [68] analyzed the mechanical and tribological properties of nylon-6 polymer composites filled with silicon nitride (Si_3_N_4_) nanoparticles. To examine the impact of various factors, including filler content and sliding speed among others, the study employed Taguchi’s design of experiments, while each parameter was tested at three different levels. The study demonstrated that composites with 4 wt. % and 16 wt. % Si_3_N_4_ nanoparticle loading showed the highest tensile strength and hardness values, respectively. However, the increase of the filler concentration beyond these levels resulted in decreased values. In the course of the research, it was noticed that the rate of degradation of the composite material due to friction increased as the speed of sliding increased. However, the incorporation of Si_3_N_4_ nanoparticles in the composite resulted in a reduction of the wear rate [55,68].

The present study involved the development of nanocomposites, by blending the ABS matrix with Si_3_N_4_ nanoparticles with the nanoparticles loading ranging from 0 wt. % to 10 wt. % to achieve a desirable level of nanofiller dispersion and distribution. The objective was to investigate the impact of Si_3_N_4_ concentration on the mechanical response of nanocomposites and gain insight into the mechanism and microstructure of the material. The thermal stability of the fabricated nanocompounds was assessed through TGA, while their chemical and elemental composition was determined using Raman and energy dispersive spectroscopy (EDS). Additionally, the impact of Si_3_N_4_ particles on the mechanical response of both the manufactured filaments and 3D-printed samples was thoroughly studied. The American Society for Testing and Materials (ASTM) standards were used to conduct the mechanical tests. Furthermore, the AFM technique was utilized to examine the surface structure of the manufactured filaments, and Scanning Electron Microscopy (SEM) was employed to analyze the structural features of all the 3D-printed samples and assess the MEX printing procedure. The demand for materials with enhanced mechanical performance in MEX 3D printing is high [69]. Therefore, the challenge of the work was to propose and analyze nanocomposites for MEX 3D printing with superior mechanical performance to broaden the applications for this technique.

## 2. Materials and Methods

**Figure 1:**
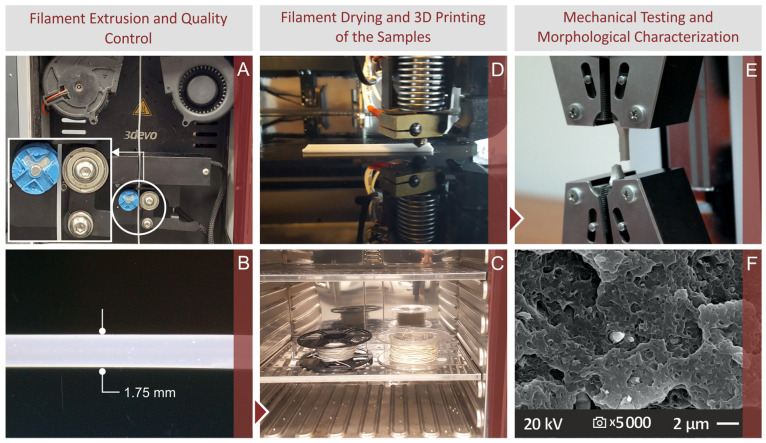
Process flow of the experimental approach, along with screenshots illustrating the specific steps taken (**A**) filament extrusion, (**B**) filament quality control, (**C**) filament drying, (**D**) 3D printing of the specimens, (**E**) tensile testing of the 3D printed samples, following the corresponding standard, (**F**) morphological analysis with SEM.

The experimental procedure used for fabricating the tested specimens and their subsequent thermomechanical and morphological characterization are shown in Figure 1 and analyzed in detail further below. Initially, the raw materials were dried and one mixture for each filler concentration was prepared, with the proper raw material quantities. The mixtures were extruded to filament compatible with the MEX 3D printing method (Figure 1A) with a two-step process, analyzed below. The produced filament underwent quality control (Figure 1C) to ensure that is properly prepared. It was dried (Figure 1C) before the 3D printing of the samples to remove any moisture from it, since the filament extrusion and the 3D printing process were not implemented on the same day. The MEX 3D printing of the samples for the mechanical tests followed (Figure 1D). Samples were fabricated in accordance with the corresponding standards for each mechanical test. Afterward, the mechanical tests were carried out (Figure 1E). Tensile test samples after their failure in the tests were examined for their morphological characteristics with SEM (Figure 1F). The side surface of the samples provided information for the quality of the 3D printing process, while the observation of the fracture surface revealed information regarding the fracture mechanism for each different nanocompound prepared and tested in the study.

### 2.1. Materials

To produce the nanocomposite samples, ABS polymer in powder (Terluran Hi-10, INEOS Styrolution, Frankfurt, Germany) was used as the matrix material. The ABS polymer displays a density of 1080 kg/m^3^ and a maximum tensile strength of 38 MPa. Nanoparticles of silicon nitride (Si_3_N_4_), measuring 760 nm in size and with a purity of 99.6%, were obtained from Nanographi (Nanographi, Ankara, Turkey).

### 2.2. Nanocomposites Preparation

**Figure 2:**
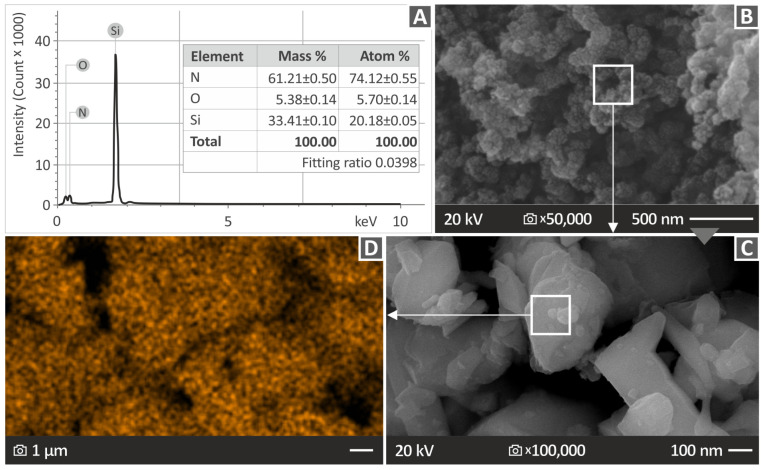
Si_3_N_4_ nanopowder investigation (**A**) analysis of EDS graph, (**B**) capturing SEM image at 50,000× magnification, (**C**) capturing SEM image at 100,000× magnification, and (**D**) EDS mapping for the Si element.

Before the preparation of the nanocomposites, the procured nanopowder underwent an evaluation through scanning electron microscopy (SEM) to determine its specific characteristics (field emission SEM JSM-IT700HR, Jeol Ltd., Tokyo, Japan). A small amount of nanopowder was placed in a glass base and it was gold-sputtered for the observation. This assessment included an investigation of its chemical and elemental composition, which was identified through the utilization of EDS measurements, as shown in Figure 2. Figure 2B,C present SEM images of the nanopowder at 50,000× and 100,000× magnifications, respectively, depicting the verification of the shape of the Si_3_N_4_ nanoparticles conducted through the examination of SEM images. Figure 2C is a 100,000× zoom of the region marked in Figure 2B. The size of the nanoparticles, according to the manufacturer, was verified by the SEM images (~760 nm). The manufacturer does not provide the shape of the specific nanoparticles. An overall asymmetrical shape with a long length compared to the width (~100 μm) was observed in the SEM images.

Figure 2A displays the identification of the elements Si and N, as evidenced by the peaks that have the most pronounced intensities. Additionally, oxygen was detected with a mass percentage of 5.38%. It is noteworthy that the existence of oxygen could be a result of moisture. The EDS mapping results for the silicon element in the Si_3_N_4_ nanomaterial are presented in Figure 2D. EDS mapping was performed in the region marked in Figure 2C. EDS mapping was performed in different areas of the scattered nanopowder under observation in the SEM to verify the results. The mapping demonstrates that the dispersion of the silicon element in the nanoparticles was almost homogeneous, with only a few voids or areas with distinct silicon concentrations. The EDS plot affirms that the nanoparticles comprise the elements predicted by the manufacturer’s formulation of the Si_3_N_4_ powder. As expected, the silicon peaks on the plot are more noticeable, suggesting a greater amount of silicon in the examined area.

The pure materials underwent a drying process for 14 h in a dryer that was set to a temperature of 60 °C. This step was taken to eliminate any residual moisture present within the materials. Five distinct blends of raw materials were prepared in separate bowls by assigning weight percentages (wt. %) of Si_3_N_4_ in concentrations of 2.0, 4.0, 6.0, 8.0, and 10.0 wt. %. This indicates that in each bowl the weight concentration of Si_3_N_4_ nanoparticles constituted a certain percentage of the nanocomposites, while the rest of the weight percentage corresponded to the existence of ABS polymer in every nanocomposite (98.0, 96.0, 94.0, 92.0, and 90.0 wt. % correspondingly). To obtain a primary dispersion of Si_3_N_4_ nanoparticles within the ABS polymer, a blender with high wattage (500 W) was utilized at room temperature (23 °C). Each mixture containing the raw materials (Si_3_N_4_ and ABS powder) at different concentrations was stirred in these bowls for 30 min with the blender operating at a speed of 4000 rpm.

Following this, the prepared mixtures were dried again as a secondary step. The production process of the filaments was conducted initially using a Noztek extruder (Noztek, Shoreham-by-Sea, UK). The Noztek extruder was used for the initial mixing of the raw materials. The filament produced by the Noztek extruder was not used for the 3D printing of the samples. The filament was shredded into pellets with a 3devo shredder (3devo B.V., Utrecht, The Netherlands). These pellets were subsequently fed into a 3devo Composer (3devo B.V., Utrecht, The Netherlands), for the final filament production. The 3devo Composer extruder used in this study is specially designed with a screw configuration suitable for combining and melting of material, i.e., in this study, the ABS matrix with Si_3_N_4_ nanoparticles, to create filaments suitable for 3D MEX printing. The filaments were produced with a 1.75 mm diameter. The 3devo Composer extruder has four heating zones along its chamber, and the temperature can be set individually in each zone to optimize the extruding procedure. The proper temperature for each zone for the ABS polymer was determined with preliminary tests and by following the corresponding literature [26,35] before the extrusion of the nanocomposites for the specific study. For all nanocompounds prepared in the study, the same extruding settings were employed for comparison purposes. Heating zone 1 was adjusted to a temperature of 220 °C, heating zones 2 and 3 were set at 230 °C, and heating zone 4 was operating at a temperature of 240 °C. The screw rotation speed was adjusted to 3.5 rpm and the fan was set at 55% to ensure proper cooling of the filaments during the manufacturing process.

The two extrusion processes described above were applied to improve the distribution of Si_3_N_4_ nanoparticles within the polymeric matrix. It is noteworthy that no additional fillers or compatibilizers were incorporated in the fabrication of the nanocomposites to assess the straight effect of Si_3_N_4_ nanoparticles on the ABS matrix. The pure ABS was also extruded into filament using the same procedure to be used as the control material for the mechanical properties of the nanocompounds.

### 2.3. Manufacture of 3D-Printed Samples

The filaments that resulted from the preceding two extrusion procedures, including pure polymer and ABS/Si_3_N_4_ nanocomposites, were employed to manufacture specimens using a Funmat HT 3D printer (Intamsys, Shanghai, China). The Intamsuite software platform was used to derive G-codes (Intamsys, Shanghai, China) utilizing the 3D-printing configuration settings that were previously established through experimentation before implementing the 3D printing process. The produced specimens were formulated to comply with the dimensional specifications outlined in the respective ASTM standards for each test type, namely ASTM D638-02a, ASTM D790-10, ASTM D6110-02, and ASTM D695 for tensile, flexural, Charpy notched impact, and compression tests, respectively. Five samples were prepared and tested for each mechanical test. Figure S1 in the Supplementary file depicts the specimen manufacturing process’ infill pattern. The rectilinear infill pattern was employed, and the raster direction alternated between +45° and −45° for successive layers. As a result, the raster direction was consistent in alternate layers of the 3D-printed object.

### 2.4. Thermogravimetric Analysis and Rheometric Examination

To assess the competence of the nanocompounds to maintain their structural integrity under high temperatures, Thermogravimetric Analysis (TGA) measurements were executed using a PerkinElmer Diamond instrument (PerkinElmer, Inc., Waltham, MA, USA) in an atmosphere consisting of N_2_. The measurements were accomplished with a gradual temperature increase of 10 °C per minute, starting from room temperature and reaching up to 550 °C. Additionally, DSC was employed using a TA Instruments Discovery Series DSC 25 (TA Instruments, New Castle, DE, USA) with a cycle range of 25–300–25 °C and a step size of 15 °C/min to examine the thermal behavior of the nanocomposites.

A DHR 20 Discovery Hybrid Rotational Rheometer from TA Instruments (TA Instruments, New Castle, DE, USA), supplemented with a parallel plate arrangement and an environmental test chamber for temperature regulation, was used to measure rheometry. The parallel plates’ diameter was 25 mm, and the measurement was made continuously, while the temperature of the test was 240 °C. These measurements evaluated the changes in the shape of a liquid sample resulting from the application of an external force. To avoid excessive heating and breakdown, each measurement point had a 20 s acquisition duration. To assess how easily the polymeric material flows through a hole with a specified diameter and length at a particular temperature and under a certain pressure, rotational rheometry experiments were combined with Melt Flow Rate (MFR) measurements. Measurements were conducted following international MFR standards (ASTM D1238-13). To ensure the material’s fluidity and avoid decomposition effects, experiments were conducted above the material’s melting point (240 °C and 230 °C, for viscosity and MFR measurements, respectively).

### 2.5. Raman Spectroscopy Measurements

To analyze the molecular bonds present in the pure ABS and the fabricated nanocompounds, measurements through Raman spectroscopy were accomplished. The Raman analysis was conducted utilizing a customized LabRAM HR Raman Spectrometer (HORIBA Scientific, Kyoto, Japan). The irritation source for Raman was obtained by employing a solid-state laser module with a 532 nm central wavelength and maximum laser output power of 90 mW. The microscope in the analysis utilized a 50× microscopic objective lens, which had a working distance of 10.6 mm and a numerical aperture of 0.5 (LMPlanFL N, Olympus, Tokyo, Japan). This objective lens was responsible to provide the excitation light on the sample and to gather the Raman signals. In parallel, a neutral density filter with a 5% transmittance was used, resulting in 2 mW of power on the sample. The resulting laser spot size was approximately 1.7 μm laterally and 2 μm axially. The Raman spectral resolution was around 2 cm^−1^ using the 600 grooves grating of the spectrometer. The purchased Raman spectral range was set between 50 to 3900 cm^−1^, providing three optical windows per point. Each measurement took a total of 50 s for acquisition when five accumulations were conducted at each point.

### 2.6. Assessment of the Manufactured Filaments

Before the 3D-printing procedure and fabricating samples, all manufactured filaments were subjected to diameter and tensile measurements, along with side surface analysis. A measuring system that employs a closed-loop control mechanism was utilized to perform a real-time measurement of the filament diameter to verify that it conforms to the desired specifications. Additionally, an electronic caliper was used to cross-check the diameter measurements. The Imada MX2 instrument (Imada Inc., Northbrook, IL, USA) was employed to conduct tensile strength evaluations. The filaments were secured in the device using standard grips, and the sample tests were accomplished at a constant rate of 10 mm/min. For each nanocomposite, a total of five filament samples were subjected to testing. Atomic Force Microscopy (AFM) measurements were employed to evaluate the morphology of the lateral surface of the filaments. In the AFM measurements, the topography of a region with a size of 10 μm × 10 μm of the side surface of the filaments was studied. Measurements were taken in different regions and an image of the surface’s 3D anaglyph was provided by the process. The surface roughness of the region under observation is calculated by the AFM device software and the Ra, Rz, and Rq surface roughness values are provided. Ra is the average profile height deviations from the mean line, Rq is the square root of the sum of the squares of the individual heights and depths from the mean line, and Rz is the difference between the minimum and the maximum peak-to-valley height of the profile within five sampling lengths. Ra and Rq are mainly related to the average roughness of the measured surface, while Rz provides an indication of the existence of high peaks and deep valleys in the measured surface. The increase in the surface roughness values can negatively affect the processability of the produced filament [15].

AFM measurements were conducted in the air using an XE7 AFM device (Park Systems, Seoul, Republic of Korea). Pictures were captured utilizing a Nanosensors NCHR cantilever (USA) equipped with a tip with a specified diameter of 10 nm, operating at a frequency of about 300 kHz. The image acquisition was conducted utilizing intermittent contact mode with a scanning rate of 0.5 Hz. A level higher than 70% of the amplitude of the oscillation that occurred naturally without any external force was maintained for the working set point during the image acquisition process.

### 2.7. Mechanical Characterization

As mentioned before, various tests were carried out based on ASTM standards to evaluate the mechanical responses of 3D-printed samples under applied forces or loads. The tests aimed to determine the ability to deform, resist fracture, and assess the overall strength and stiffness of the samples, and the impact of Si_3_N_4_ nanoparticles on the mechanical response of nanocomposites. All the tests were carried out under standard environmental conditions (23 °C and 55% humidity), along with their respective parameters, as presented in Figure S1 in the Supplementary file. For each analyzed ABS/Si_3_N_4_ nanocomposite, a total of five samples were manufactured and examined through testing. The equipment and parameters used are as follows:

- Tensile tests: Imada MX2 (Imada Inc., Northbrook, IL, USA), 10 mm/min strain rate, standard grips.
- Compression tests: Instron KN1200 (INSTRON, Norwood, MA, USA) at a rate of 1.3 mm/min.
- Flexural tests: Imada MX2 (Imada Inc., Northbrook, IL, USA), 10 mm/min strain rate, 52 mm support span.
- Impact tests: Terco MT-220 (TERCO, Kungens-Kurva, Sweden), Charpy, notched, hammer release height 367 mm.
- Microhardness: InnovaTest 300 (INNOVATEST, Maastricht, The Netherlands), Vickers, 200 gF, 10 s.

### 2.8. Morphological Characterization of 3D-Printed Samples

To thoroughly analyze the morphology of the fracture and side surfaces of the manufactured 3D printing samples, SEM was performed using a field emission SEM (JSM-IT700HR, Jeol Ltd., Tokyo, Japan) at 20 kV in a high vacuum environment. SEM images were taken on tensile specimens after they failed in the experiment. Samples from each different nanocompound prepared herein were examined. The side surface observations aimed to evaluate the 3D printing quality of the samples, while the fracture surface inspection aimed to study the fracture mechanism in each sample. Additionally, these observations aimed to locate agglomerations of the Si_3_N_4_ filler in the nanocompounds.

The gold-sputtered samples were imaged at various magnifications and different regions using SEM. Furthermore, the chemical composition of elements present in both the pure polymer and all nanocomposites was confirmed through EDS analysis.

EDS mapping was performed in regions of the fracture surface of one 3D-printed sample of each one of the nanocomposites prepared in the study. EDS mapping also aimed to identify which parts of the observation area refer to nanoparticles.

## 3. Results

### 3.1. Thermal Properties Assessment with Thermogravimetric Analysis and Differential Scanning Calorimetry

**Figure 3:**
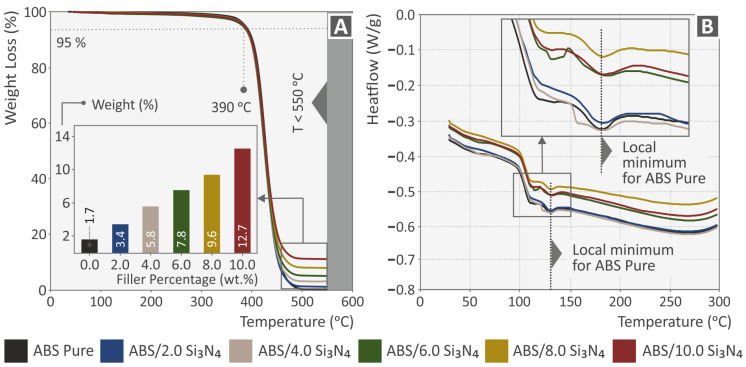
The decomposition of the pure ABS and ABS/Si_3_N_4_ nanocompounds is illustrated in (**A**) TGA graphs and (**B**) heat-flow curves, as a function of temperature.

Figure 3A displays the TGA diagrams of the pure ABS and the examined nanocomposites, illustrating the weight loss compared to the temperature. It can be observed from Figure 3 that the ability of the ABS material to resist thermal decomposition is not impacted by the addition of Si_3_N_4_ nanoparticles. Thus, the reduction in weight of all nanocomposites initiates at 390 °C, which is comparable to the weight loss temperature of pure ABS. The temperature at which the weight loss began was noticeably greater than the temperature employed in the extrusion processes, i.e., filament manufacturing and the MEX 3D printing. In the inset bar charts presented in Figure 3A, the remaining weight after the completion of the TGA is presented. As shown, this weight is in agreement with the filler concentration in all nanocompounds. Additionally, Figure 3B depicts the heat flow curves for the designated ABS matrix and the manufactured nanocompounds. As can be observed, the trends of all materials are comparable, and the relaxation peaks are also clearly visible.

### 3.2. Rheometric Measurements

**Figure 4:**
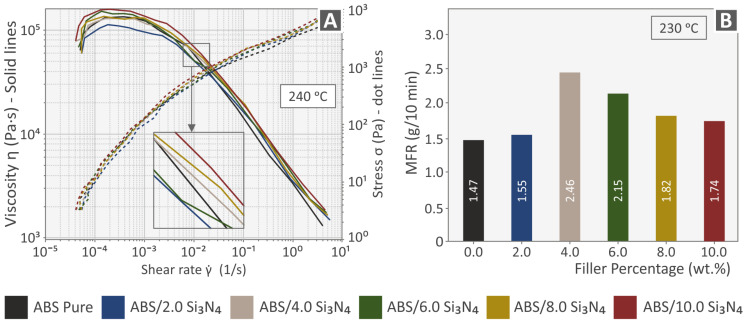
Rheometric measurements of the pure polymer and the Si_3_N_4_ composites: (**A**) Viscosity and Stress vs. Shear rate, (**B**) Melt Flow Rate vs. filler content.

Figure 4A presents the results obtained from the rheological measurements and, more specifically, the viscosity and stress of the samples as a function of shear rate, plotted on logarithmic axes. Viscosity generally decreased with the shear rate for all samples, indicating a non-Newtonian, shear-thinning, or pseudoplastic behavior. This behavior has previously been reported in the literature, both for pure ABS and ABS-based composites [70,71,72,73]. A shear-thinning nature is commonly preferred for printable materials. First, it allows them to flow smoothly from the printing element, which induces high shear stress to the material, onto the print bed. Second, the material needs to retain its extrudate shape after printing, namely absent any shear forces [74]. At low shear rates, all samples exhibited quasi-Newtonian behavior. For the samples filled with Si_3_N_4_ the quasi-Newtonian region became shorter. Although the additive did not substantially alter the rheological behavior of the matrix under shear stress, higher percentages of Si_3_N_4_ could be associated with shorter Newtonian regions and higher viscosity values [70,71]. The latter can be attributed to the polymer matrix inducing viscous forces that align the particles of the additive in shear bands, thus causing the material to resist flow under shear stress. The viscosity values of the filled samples at different shear rates were close to that of pure ABS, which indicated that there was no chemical interaction between the filler and the matrix. The curves of the samples containing Si_3_N_4_ at 2.0, 4.0, 6.0, and 8.0% wt. percentages seemed to converge at higher shear rates. This implies that under higher shear stress the rheology is greatly influenced by the molecular orientation of the ABS matrix [71]. The results obtained from the melt flow index measurements are presented in Figure 4B in the form of the melt flow rate (MFR in g/10 min) as a function of filler weight percentage. Flow rate generally increased with the addition of fillers in comparison to the pure polymer, with its highest value measured at a filler loading of 4 wt. %. Higher loadings seemed to gradually decrease the composite material flow rate which is consistent with the rheological measurements.

### 3.3. Raman Spectroscopy Measurements

**Figure 5:**
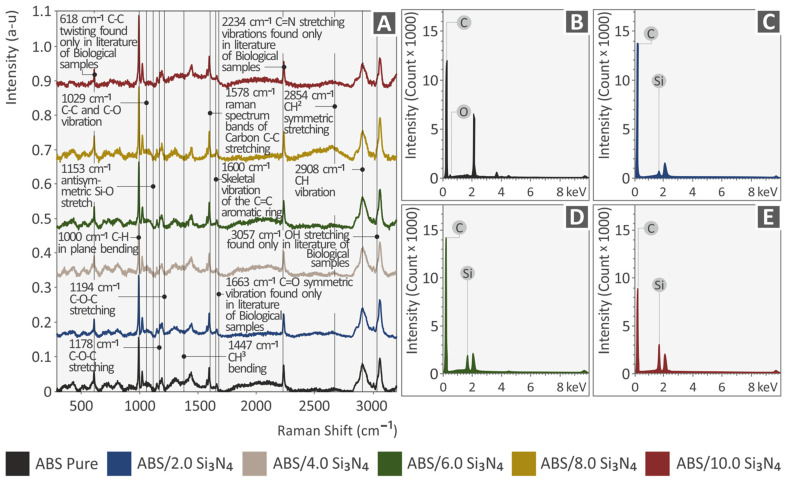
(**A**) Raman spectra for all samples tested: EDS analysis for ABS pure and ABS/Si_3_N_4_ nanocomposites: (**B**) pure ABS, (**C**) ABS/2.0 Si_3_N_4_, (**D**) ABS/6.0 Si_3_N_4_, (**E**) ABS/10.0 Si_3_N_4_.

Figure 5A presents the Raman signals of all the samples used in this study. It can be observed that all the Raman lines come from pure ABS. The related Raman signals are shown in the following Table 1 and are validated by the literature. The addition of Si_3_N_4_ showed some differences in the Raman spectrum, which were only found when normalization with the ABS Pure Raman spectrum was performed. As can be seen, all Raman lines had a drop linearly related to the increase of the Si_3_N_4_ concentration. The related Raman lines are presented in the following Table 2. Figure 5B–E display the EDS curves, which offer elemental examination for the four nanocomposites studied. It is noticeable that carbon is detected in all samples, with the highest percentages found in ABS/Si_3_N_4_ 2.0 wt. % and ABS/Si_3_N_4_ 6.0 wt. %. The nanocomposite with the highest percentage of Si_3_N_4_, on the other hand, displays the lowest amount of carbon. Si was located in all nanocompounds as expected and no unwanted elements were identified in the nanocompounds through EDS.

### 3.4. Filament Assessment

**Figure 6:**
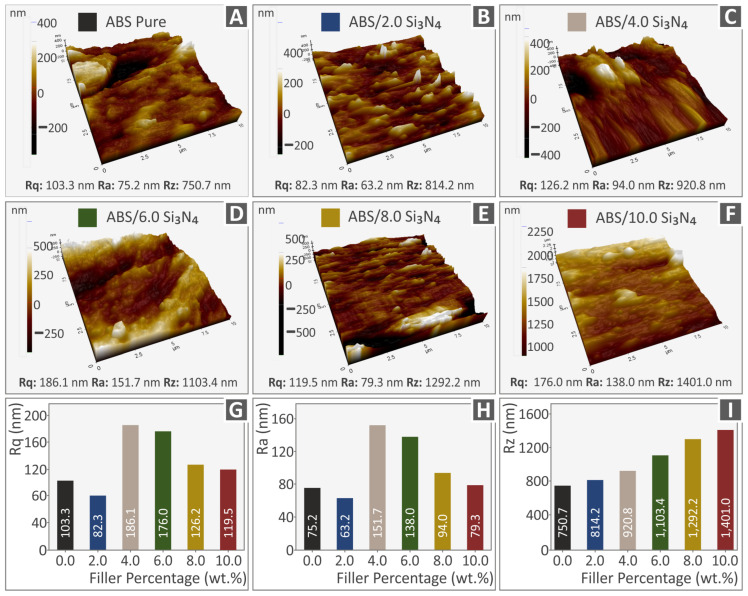
Pictures of the tested filaments’ side surfaces using AFM for (**A**) pure ABS, (**B**) ABS/1 wt. % Si_3_N_4_, (**C**) ABS/2 wt. % Si_3_N_4_, (**D**) ABS/4 wt. % Si_3_N_4_, (**E**) ABS/6 wt. % Si_3_N_4_, and (**F**) ABS/8 wt. % Si_3_N_4_, and graphs showing the relationship between the Si_3_N_4_ content and the surface roughness parameters in the nanocompounds (**G**) Rq, (**H**) Ra, (**I**) Rz.

AFM was conducted to investigate the morphology of the side surfaces of all produced filaments. As shown in Figure 6, the majority of the filaments display greater surface roughness measurements when compared with the pure ABS samples. The addition of Si_3_N_4_ in the ABS matrix has a noticeable effect, causing a substantial rise in the roughness of the surface and a notable alteration in the filament morphology. Graphs in Figure 6G–I present the correlation between the Si_3_N_4_ content in the nanocompounds and the three surface roughness measurements (Rq, Ra, and Rz). The ABS/Si_3_N_4_ 2.0 wt. % nanocomposite shows a minor decrease in the Rq and Ra roughness values (Figure 6G,H). A substantial increase is observed beyond this point, indicating up to 6.0 wt. % Si_3_N_4_ and then the Ra and Rq values decrease again, still maintaining higher values than the pure ABS. Furthermore, Figure 6I illustrates a nearly linear and gradual rise in the Rz surface roughness values with increasing the Si_3_N_4_ additive. Such measurements indicate that the average surface roughness is increased by the addition of Si_3_N_4_ in the matrix material. The increase of Rz indicates that as the filler concentration increases in the nanocomposites higher peaks and deeper valleys are formed on the side surface of the produced filament. The filament’s roughness value increases due to imperfections on the surface, which can also negatively affect how easily it can be processed during the 3D printing procedure [15].

**Figure 7:**
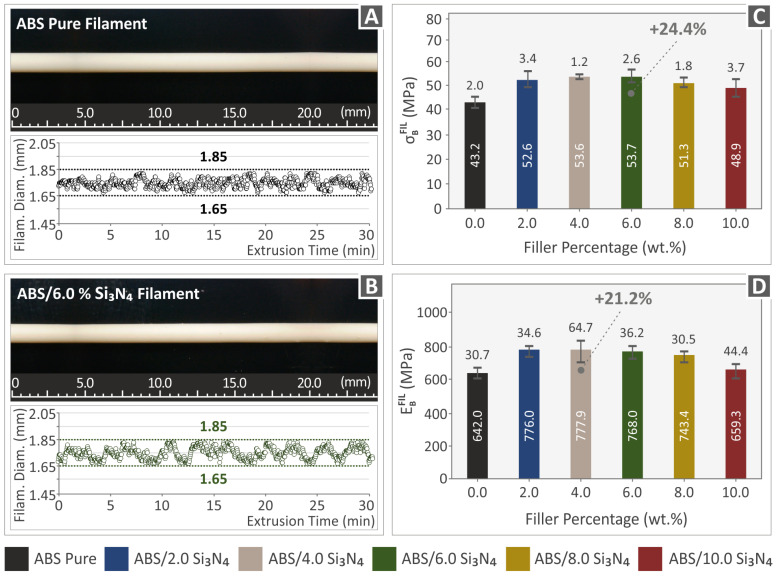
Pictures of the extruded filament segments and their real-time diameter measurements for (**A**) ABS pure filament, (**B**) ABS/6.0% Si_3_N_4_ filament, (**C**) tensile tests results, and (**D**) modulus of elasticity results of the manufactures filaments.

As previously stated, the filaments under examination were produced through the utilization of a 3devo composer extrusion machine (3devo B.V., Utrecht, The Netherlands) equipped with a closed-loop system for controlling the filament diameter. Through the real-time measurement of the filament diameter and by making automatic adjustments to the extrusion procedure within tolerable limits, the integrated system can produce a uniformly sized filament with an accurate diameter. Two segments of the produced filaments, chosen randomly, are displayed in Figure 7A,B. These images were captured using an optical stereoscope model name OZR5 (OZR5, KERN & SOHN GmbH, Albstadt, Germany). Additionally, the figures show real-time measurements of the diameters of pure ABS and ABS/Si_3_N_4_ 6.0 wt. % nanocompounds. It is evident from the observation that the measurements of the filament’s diameter show a variation (200 µm), which is considered suitable for MEX 3D printing. This confirms the sufficiency of the experimental methodology and the meticulous identification of the control factors. The images captured through optical stereoscopy (Figure 7A,B) revealed filaments with a seamless surface and no imperfections.

Figure 7C displays the findings of the filament tensile tests, indicating a significant improvement in the tensile strength for all Si_3_N_4_ concentrations. The maximum increase of 24.4% was achieved by the 6.0 wt. % Si_3_N_4_ nanocompound. In Figure 7D, it can be observed that the incorporation of Si_3_N_4_ nanoparticles influenced the stiffness of the manufactured filaments. The ABS/Si_3_N_4_ 4.0 wt. % nanocompound demonstrated the highest stiffness, exhibiting a 21.2% increase compared to the ABS matrix. It should be noted that the filament tensile tests were not conducted according to a standard so the findings cannot be correlated with tests on dogbone samples. The tensile tests on the filament were carried out to have an insight into the performance of the solid (not 3D-printed) nanocompounds and evaluate the effect of the filler concentration in the nanocomposites and how this effect differs in the 3D printed parts in which the 3D printing process affects their performance [35].

### 3.5. Mechanical Examination of the 3D-Printed Samples

**Figure 8:**
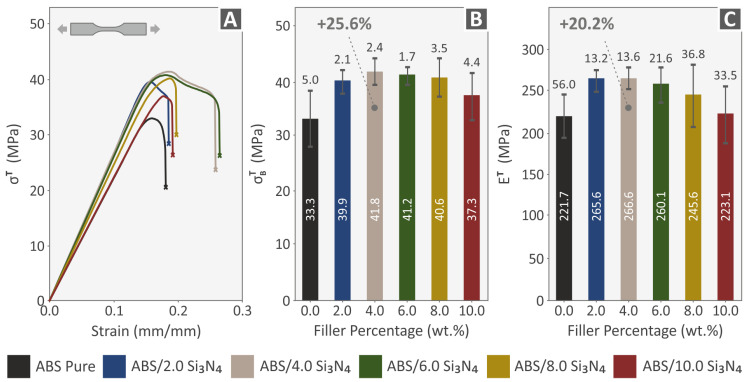
Tensile test results of all 3D-printed samples: (**A**) graphs of tensile stress vs. calculated strain from one of the five 3D-printed specimens for each nanocomposite (randomly selected), (**B**) tensile strength results, and (**C**) tensile modulus of elasticity results.

Following the assessment of the produced filaments, the 3D-printed samples were exposed to tensile testing under the ASTM D638-02a standard to analyze their mechanical behavior. A graph of tensile stress (MPa) versus calculated strain (mm/mm) was created for one representative sample of each nanocomposite material and the pure ABS polymer, as shown in Figure 8A. Figure 8B illustrates the average tensile strength at the point of failure (in MPa) for every material, plotted against the filler percentage. Additionally, the corresponding tensile modulus of elasticity (in MPa) for all filler percentages assessed, relative to the unfilled ABS polymer, is depicted in Figure 8C. The experimental findings show that all samples with varying additive concentrations exhibit improved tensile properties when contrasted with the unfilled ABS polymer. Notably, the 4.0 wt. % Si_3_N_4_ sample displays the most significant enhancement in terms of both the tensile strength and stiffness, with increments of 25.6% and 20.2%, correspondingly. These findings suggest that there is a threshold of 4.0 wt. % Si_3_N_4_ where the mechanical properties reach their maximum values and further addition of Si_3_N_4_ does not result in improvement in the mechanical performance of the nanocompounds.

**Figure 9:**
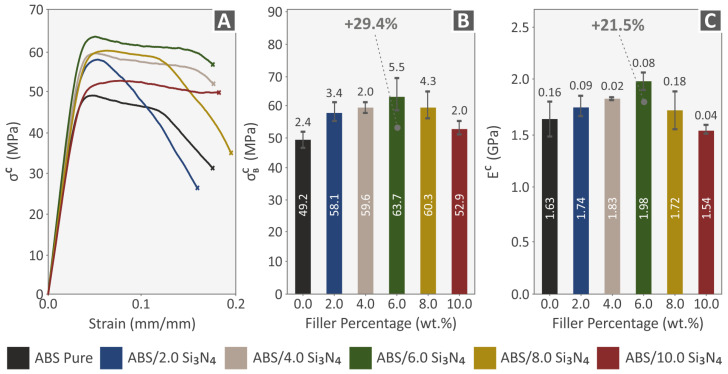
Compression tests of all 3D-printed samples: (**A**) graphs of compression stress vs. calculated strain from one of the five printed specimens for each nanocomposite (randomly selected), mean values and deviations of (**B**) compression strength results, and (**C**) compression modulus of elasticity results.

**Figure 10:**
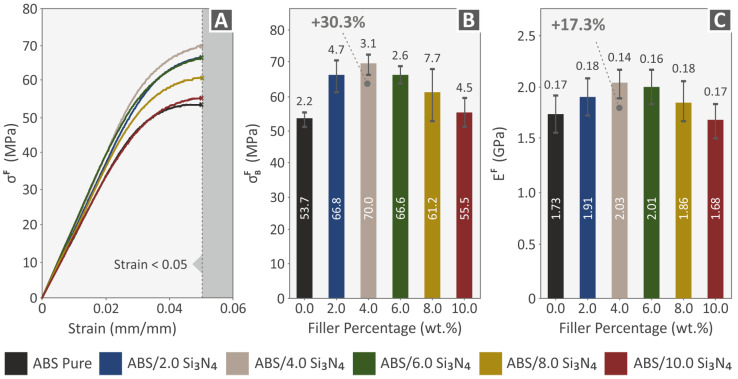
Flexural tests of all 3D-printed samples: (**A**) graphs of flexural stress vs. calculated strain from one of the five printed specimens for each nanocomposite randomly selected (experiment termination: 5% strain, according to the ASTM D790 standard, mean values and deviations (**B**) flexural strength results, and (**C**) flexural modulus of elasticity results.

Figure 9 summarizes the compression properties of samples made from pure ABS and ABS/Si_3_N_4_ nanocomposites containing Si_3_N_4_ filler loadings ranging from 2.0 to 10.0 wt. %. The compression strength-stress curves for the various samples tested are depicted in Figure 10A, while the average compression strength is illustrated in Figure 9B. The average compression modulus of elasticity for the 3D-printed specimens is presented in Figure 9C. Upon analyzing the results of the compression tests (Figure 9), it was observed that the compression strength and compression modulus of elasticity exhibited a comparable pattern to the tensile tests, with the compression strength increasing as the concentration of the additive increased. The compression strength reached the highest values on the 4.0 wt. % Si_3_N_4_ nanocompound, reaching a 29.4% increase compared to pure ABS. The highest improvement in the compression modulus of elasticity was achieved by the 6.0 wt. % Si_3_N_4_ nanocompound, reaching a 21.5% increase compared to pure ABS.

The flexural properties of pure ABS and ABS/Si_3_N_4_ nanocomposite specimens are summarized in Figure 10. Figure 10A shows stress–strain curves for the different samples tested for flexural strength, while Figure 10B displays the average flexural strength and Figure 10C shows the average flexural elasticity modulus of the 3D-printed specimens. The flexural strength mean values were determined using the ASTM D790-10 standard and calculated at a maximum strain of 5% due to no breakage in the specimens during testing, following the standard’s instructions. All nanocomposites show increased flexural strength values, except for the 10.0 wt. % Si_3_N_4_ nanocomposite, which has a minor reduction in the flexural modulus of elasticity. There is a noticeable increase in flexural strength up to 4.0 wt. % nanocompound which achieved a supreme flexural strength of 70.0 MPa, 30.3% greater than pure ABS. The same pattern can be observed in the flexural modulus of elasticity, with the nanocomposite containing 4.0 wt. % exhibiting the optimal value of 2.03 GPa, presenting a remarkable rise of 17.3% in comparison to ABS material.

**Figure 11:**
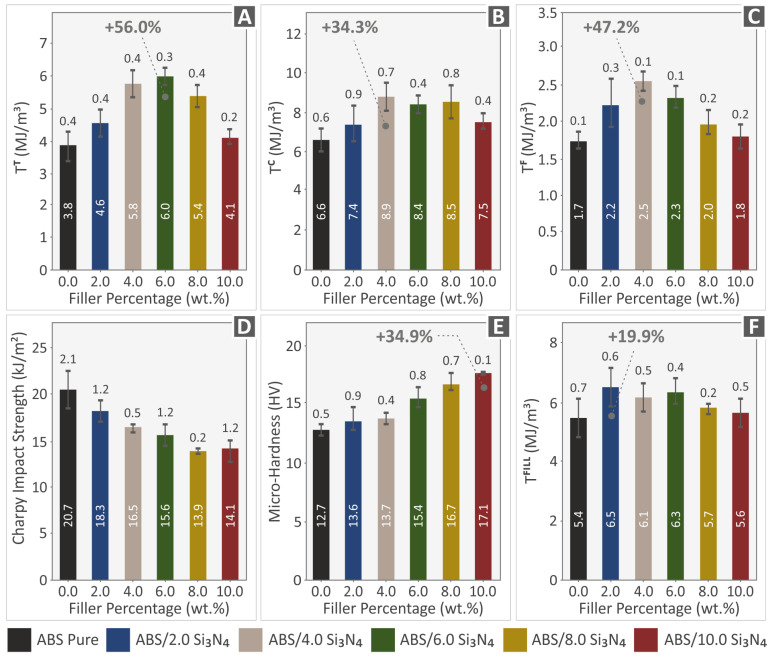
Results (mean values and deviation) from (**A**) Tensile toughness tension for all fabricated specimens, (**B**) Compression toughness tension for all fabricated specimens, (**C**) Flexural toughness tension for all fabricated specimens, (**D**) Impact strength, (**E**) Vickers microhardness, and (**F**) Tensile toughness tension for all fabricated filaments.

Figure 11A–C,F present information on the toughness values (MJ/m^3^) of various materials, in both filament and specimen forms, obtained during mechanical testing. After analyzing the stress–strain graphs, the energy absorbed by the materials during testing was quantified, as an integral of the corresponding stress vs. strain curves, to determine their tensile, compression, and flexural toughness values. This information is useful for understanding the fracture mechanism and can be applied to develop a “safe-fail” process for different applications. Based on the information presented in Figure 11A–C,F, it can be concluded that the nanocomposites have higher levels of tensile, compression, and flexural toughness in comparison to the ABS polymer.

Figure 11D,E display the outcomes of the impact tests and the Vickers microhardness examinations respectively. These figures show the average Charpy impact strength (kJ/m^2^), and the Vickers microhardness values (HV) computed for all the materials examined, corresponding to the filler percentage (%). The impact strength of the nanocomposites showed a clear pattern based on the amount of Si_3_N_4_ present, unlike other mechanical characteristics. The incorporation of Si_3_N_4_ filler into the polymeric matrix negatively impacted the material’s mechanical response, resulting in reduced impact strength in all nanocomposites tested when associated with the pure ABS polymer. Nonetheless, the obtained microhardness outcomes showed a clear improvement in all tested nanocomposites, with higher values compared to the polymeric matrix. As the concentration of the additive was increased in the ABS/Si_3_N_4_ wt. % nanocomposites, there was a gradual increase in microhardness. Notably, nanocomposites containing 10.0 wt. % Si_3_N_4_ established a remarkable enhancement in their microhardness, with a value of 17.1 HV, demonstrating a 34.9% increase in comparison to the ABS material.

### 3.6. Morphological Characterization of the 3D-Printed Samples

**Figure 12:**
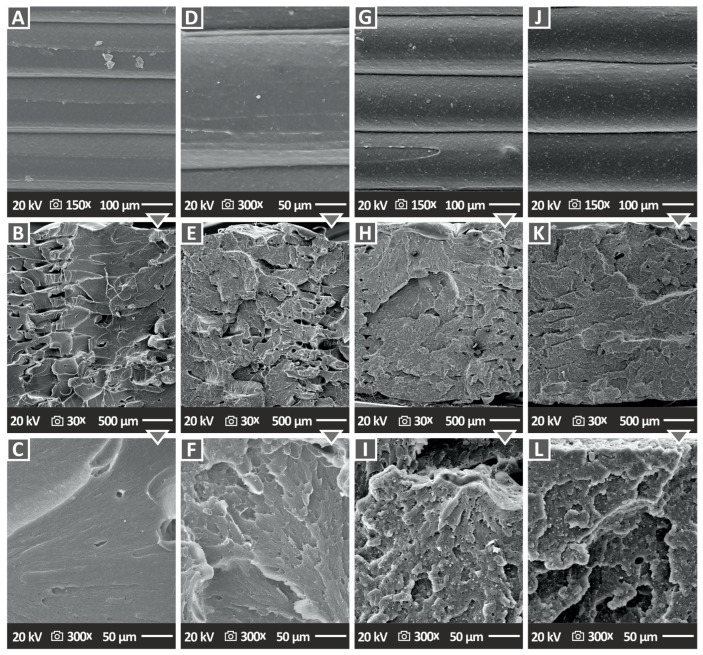
SEM pictures for (**A**) side surface of pure ABS specimen at 150× magnification, (**B**) fracture surface of pure ABS specimen at 30× magnification, (**C**) fracture surface of pure ABS specimen at 300× magnification, (**D**) side surface of ABS/2.0% Si_3_N_4_ specimen at 300× magnification, (**E**) fracture surface of ABS/2.0% Si_3_N_4_ specimen at 30× magnification, (**F**) fracture surface of ABS/2.0% Si_3_N_4_ specimen at 300× magnification, (**G**) side surface of ABS/4.0% Si_3_N_4_ specimen at 150× magnification, (**H**) fracture surface of ABS/4.0% Si_3_N_4_ specimen at 30× magnification, (**I**) fracture surface of ABS/2.0% Si_3_N_4_ specimen at 300× magnification, (**J**) side surface of ABS/6.0% Si_3_N_4_ specimen at 150× magnification, (**K**) fracture surface of ABS/6.0% Si_3_N_4_ specimen at 30× magnification, (**L**) fracture surface of ABS/6.0% Si_3_N_4_ specimen at 300× magnification.

Later on, the morphology of the 3D-printed specimens was analyzed through SEM imaging, which examined both the fractured surfaces and sides of the specimens. Figure 12A,D,G,J exhibit pictures of the side surface of four samples—pure ABS material and three types of nanocomposites (ABS/Si_3_N_4_ 2.0 wt. %, ABS/Si_3_N_4_ 6.0 wt. % and ABS/Si_3_N_4_ 10.0 wt. %) at a magnification of 150× (and 300× for one sample to further evaluate the 3D printing structure). These images indicate that all specimens have flawless layer interfusion without any imperfections or empty spaces and uniform layer shape. This observation confirms that the 3D printing process used produced high-quality samples. Moreover, this confirms that pure ABS and the tested nanocompounds are compatible with the applied setting parameters and that the filler loading does not impact the layer interfusion.

The fractured surfaces of the specimens were captured using SEM. Figure 12B,E,H,K displays the fracture surface at a magnification of 30×, while Figure 12C,F,I,L does so at a magnification of 300×. In Figure 12B, microvoids were detected in the pure ABS sample, primarily near the edges of the specimen. These microvoids are normal in the 3D printing structure as a result of the MEX 3D printing process that builds the object layer, even at a 100% infill ratio [15]. When examining the pure ABS through the SEM image at a magnification level of 300× (Figure 12C), the resulting image shows no deformation in the filament strands. The SEM images of the fractured surface of the ABS/Si_3_N_4_ nanocompound containing 4.0 wt. % filler is displayed in Figure 12H,I at two magnification levels. These images reveal the existence of microvoids and microporosity, which provides evidence for the absorption of moisture in the specimen [84,85]. It is worth noting that these voids are not present in the filament and, therefore, they do not affect the overall efficiency. In the nanocompounds higher deformation levels are presented compared to the pure ABS, indicating a more ductile fracture mechanism in the samples. This finding is in agreement with the corresponding stress vs. strain graphs (Figure 8A).

**Figure 13:**
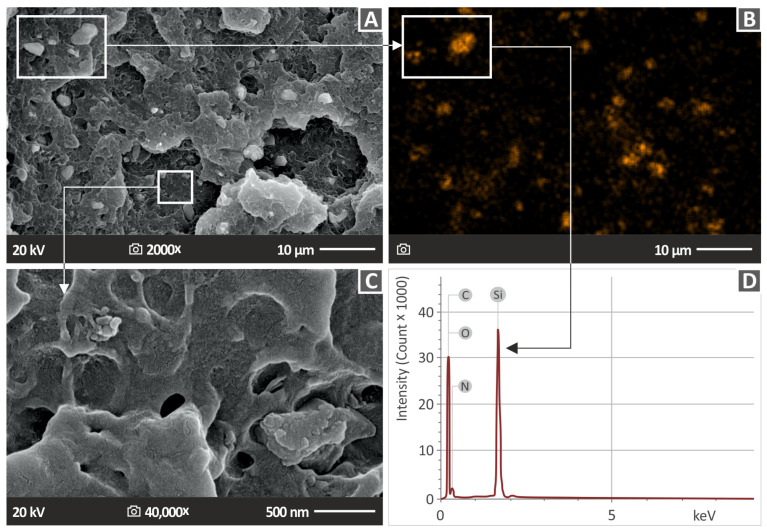
SEM pictures of the side surface of (**A**) ABS/10.0% Si_3_N_4_ at 2000× magnification, (**B**) EDS mapping for the Si element in the nanocomposite, (**C**) ABS/10.0% Si_3_N_4_ at 40,000× magnification, and (**D**) EDS analysis for ABS/10.0% Si_3_N_4_ acquired from a Si_3_N_4_ nanoparticle-rich region.

Figure 13 examines the distribution of the nanoadditive in the ABS polymer and identifies any potential agglomerations. Figure 13A shows an SEM image of the ABS/Si_3_N_4_ 10.0 wt. % nanocomposite, which revealed clusters of Si_3_N_4_ nanoparticles. The surface’s microporosity, as shown in Figure 13C at 40,000× magnification of the area shown in Figure 13A, suggests that the presence of a nanovoid could be owed to moisture absorption [15]. Figure 13B shows EDS mapping for the Si element in the entire region shown in Figure 13A. The distribution of the Si element in the EDS mapping verifies that the particles shown in Figure 13A are indeed Si_3_N_4_ nanoparticles. A region with higher Si content indicates that the nanoparticles agglomerated in this region.

Agglomerations were located only in the ABS/Si_3_N_4_ 10.0 wt. % nanocomposite and justify the decrease in the mechanical properties of this specific nanocompound. Apart from the agglomeration area, the distribution of the Si element was rather uniform, indicating a rather well distribution of the nanoparticles in the matrix with the process followed for their development. Figure 13D displays the corresponding EDS graph obtained from the area of agglomeration in the 10 wt. % filler loading specimen. The EDS graph showed a significant silicon peak, suggesting a substantial amount of this element is present in the observation area. The detection of carbon and nitrogen elements was validated by the EDS analysis, while a notable oxygen intensity was also detected, which might be a significant factor for the presence of microvoids and likely originated from the Si_3_N_4_ nanopowder [15].

## 4. Discussion

**Figure 14:**
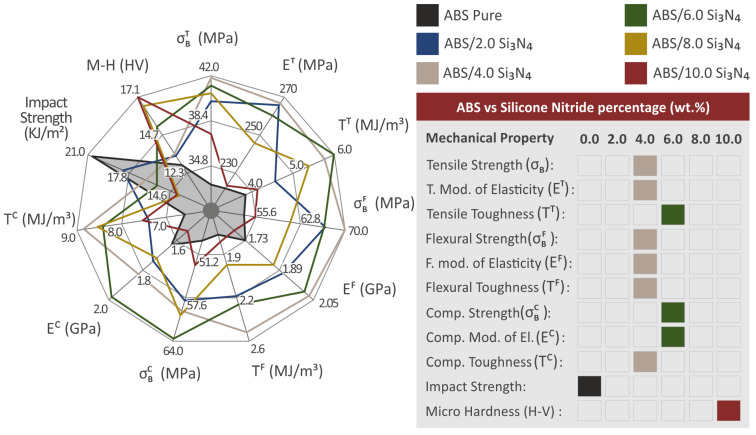
The spider diagram on the left shows the mechanical characteristics of the examined 3D-printed samples. The gray region depicts the performance of the pure ABS that was manufactured and tested for research to serve as a benchmark for assessing the enchantment achieved. The materials that demonstrate the highest mechanical response in each experiment are listed in the table on the right.

Figure 14 provides an overview of the mechanical tests performed on the examined nanocompounds and the pure ABS polymer. The addition of nanoparticles generally improved the properties of a material. One of the main factors affecting the mechanical performance of the nanocomposites is the interaction between the filler and the matrix, as well as the effect of the filler on the rheological properties of the matrix. Additionally, the mechanism that leads to mechanical performance improvement can also be influenced by the chemical responses occurring at the interface between the nanoparticles and the matrix [86,87,88].

The 4.0 wt. % nanocompound had the highest mechanical response in most of the experiments. As a result, it can be concluded that this loading is the optimum one among the nanocompounds prepared and tested herein. In particular, the ABS/Si_3_N_4_ 4 wt. % specimens exhibit the highest improvement in their flexural properties, along with notable enhancements in the tensile strength (σ_Β_) and stiffness. It is noteworthy that the majority of the mechanical tests exhibit their highest values as mentioned at 4.0 wt. %, indicating that the mechanical properties enhancement is linked to the Si_3_N_4_ concentration up to this point. Further increasing the Si_3_N_4_ reduces the mechanical properties, showing that saturation of the Si_3_N_4_ in the ABS matrix occurs at higher concentrations. The exact percolation threshold was not located, as it was not within the scope of the current study. Still, this is an indication that it was reached, and this negatively affects the mechanical properties of the nanocomposites [89,90]. It should be noted though that even the nanocomposites with the highest loading of 10 wt. % achieved higher values in their mechanical properties in most of the experiments compared to the unfilled ABS polymer.

Additionally, the tensile toughness (Τ^Τ^) increases significantly up to a 6.0 wt. % filler loading, reaching a value of 56 %. However, microhardness demonstrates a continuous and favorable increase up to a 10 wt. % filler loading, reaching 34.9 %. Among all the properties that the addition of Si_3_N_4_ nanoparticles had an impact on, only impact strength shows a decrease in mechanical response. It seems that the amount of Si_3_N_4_ has a direct impact on improving the resulting nanocompounds, except for their capabilities under impact loadings.

By examining the microstructure of the 3D-printed samples from its side surface, important information such as the thickness of each printed layer and the quality of the 3D printing can be determined. This analysis can also reveal the characteristics of the interfaces between the layers and the fusion between them. SEM images show that increasing the concentration of filler in the ABS matrix does not result in more voids on the surface of the 3D-printed samples when compared to pure ABS. However, it is worth mentioning that the specimens containing 4.0 wt. % ABS/Si_3_N_4_ have significantly larger microvoids compared to those with higher filler content. Still, their mechanical performance was the most enhanced one in most tests, showing that in this case, such imperfections in the 3D printing structure do not negatively affect the mechanical response of the nanocomposites. As noted above, such imperfections in the structure of parts made with the MEX 3D printing process are expected in the samples.

It is important to mention that TGA analysis confirms that the processing temperatures used during the MEX method do not cause any damage to the materials utilized, which could negatively impact the 3D printing process overall and the mechanical performance of the samples made with the prepared nanocomposites. It is also important to note that the mechanical properties of the ABS polymer were upgraded at nearly every concentration of Si_3_N_4_, with the greatest reinforcement observed at a loading of 4.0 wt. %. This indicates that the incorporation of Si_3_N_4_ nanoparticles into the ABS matrix leads to a clear and consistent improvement of the polymer’s strength.

No study so far in the literature investigates similar nanocomposites for their mechanical performance to the authors’ best knowledge. Therefore, the findings of the current work cannot be evaluated with the literature. As presented in the literature review section, overall research in ceramics additives as reinforcement agents for polymers in 3D printing is still rather limited. In MEX 3D printing titanium nitride (TiN) has been introduced in different polymeric matrices as a reinforcement agent. In the polylactic acid (PLA) matrix, a 43.4% improvement in the tensile strength compared to the pure polymer was achieved [91]. The corresponding improvement in the polycarbonate (PC) polymer was 35.5% [92], in the polyamide 12 (PA12) 45.5% [93], and, finally, in the ABS polymer 18.1% [15]. These results show that the same ceramic additive had a different effect in different polymeric matrices. The reinforcement effect was lower in the ABS polymer than in all the other matrices. In the current study a 25.6% improvement in the tensile strength was achieved, showing that the Si_3_N_4_ nanoparticles achieved a higher reinforcement effect on the ABS matrix than TiN.

It should be noted that differences are expected when comparing the findings of the current study with the literature, attributed to different material grades, process parameters, and methodology followed, among others. The results of the filament tensile tests are in agreement with the literature [15]. Such tests, as mentioned, were carried out mainly for a qualitative assessment of the effect of the introduction of the filler than a quantitative one. In the 3D-printed parts, the 3D printing setting values can significantly affect their mechanical performance. The introduction of the additives affected the rheological characteristics of the nanocomposites compared to the pure ABS polymer and this also has an effect on the performance of the 3D-printed samples.

In conclusion, it is worth noting that the suggested approach of incorporating Si_3_N_4_ nanoparticles into the ABS matrix for polymer reinforcement does not result in a significant cost increase for the production of the 3D parts. The sole expense in the procedure is the mixing of raw materials, which can be considered minimal, particularly in applications on an industrial scale. The inclusion of Si_3_N_4_ nanoparticles incurred an additional cost for the materials used. ABS raw material is priced at 0.35 €/kg for orders exceeding 1000 kg, while the resulting filament costs roughly 25 €/kg. This indicates that the main cost of the commercial filament is the cost of the process and not the cost of the raw materials. Si_3_N_4_ nanoparticles have a price of around 0.4 €/g when used for laboratory-scale experiments, but this cost can be decreased meaningfully for industrial-scale use. For laboratory-scale experiments, the cost of ABS is around 10 €/kg, which is equal to 0.01 €/g. The optimal mechanical responses were observed with a 4.0 wt. % loading of the nanocompound, which results in a cost of 0.016 €/g for the Si_3_N_4_ particles and a total cost of 0.026 €/g for laboratory-scale use (0.01 €/g + 0.016 €/g). The increase in raw material expenses is notable, but it has a minimal impact on the total cost of the filament manufacturing procedure.

## 5. Conclusions

The study’s hypothesis was confirmed as the results indicated that adding Si_3_N_4_ nanoparticles improved the mechanical properties of the polymeric matrix. The majority of the mechanical characteristics displayed notable enhancements at the weight concentration of 4.0 wt. % Si_3_N_4_ compared to the mechanical properties of the pure ABS polymer. The tensile toughness showed the most significant improvement, with an increase of 56% compared to pure ABS. The flexural properties also showed enhanced values, with the flexural strength increasing by 30.3%, flexural toughness by 47.2%, and flexural modulus of elasticity by 17.3%. Moreover, the tensile strength and tensile modulus of elasticity showed improvements of 25.6% and 20.2%, respectively. The compression properties also displayed enhancements, with the compression strength increasing by 29.4%, compression toughness by 34.3%, and compression modulus of elasticity by 21.5%. The microhardness of the nanocomposites displayed a gradual improvement with an increase in the Si_3_N_4_ content, with a maximum enhancement of 34.9%. The hardness of the ceramic nanoadditive can be credited with causing this enhancement.

According to the thermogravimetric analysis, the thermal stability of the thermoplastic polymer was not significantly altered by the incorporation of the Si_3_N_4_ nanoparticles. This finding supports the argument that the materials examined are compatible with the methodology employed, as well as the temperatures and conditions utilized in all stages of the procedure. The highest temperature used in the 3D printing procedure is significantly lower than the temperature at which weight loss begins, which is 390 °C. Raman spectroscopy analyses revealed that there were no changes detected in the chemical bonds of the polymeric matrix, nor were any chemical reactions observed between ABS and Si_3_N_4_ nanoparticles. The study findings can be easily scaled up for industrial use for the production of nanocomposites with the ABS matrix with improved mechanical performance, utilizing an easy, cost-effective approach. In future work, the required steps for the industrialization of the process can be investigated, along with the exact percolation threshold of the additive in the matrix. Additionally, the 3D printing parameters can be further analyzed and optimized to maximize the reinforcement effect of the Si_3_N_4_ additive in the ABS polymer, further increasing the merit of this study’s results.

## Figures and Tables

**Table 1:** Major Raman peaks of ABS pure detected and their related assignments.

Wavenumber (cm^−1^)	Raman Peak Assignment
618	C-C twisting is found only in the literature on biological samples [75]
1000	C-H in-plane bending [76]
1029	C–C and C-O vibration [77]
1153	Antisymmetric Si-O- stretch [78]
1178	C-O-C stretching [76]
1194	C-O-C stretching [79]
1447	CH_3_ bending [76,79,80]
1578	Raman spectrum bands of Carbon C-C stretching [81]
1600	Skeletal vibration of the C=C aromatic ring [82,83]
1663	C=O symmetric vibration found only in the literature of biological samples [75]
2234	C≡N stretching vibrations are found only in the literature of biological samples [75]
2854	CH_2_ symmetric stretching [77]
2908	CH vibration [77]
3057	OH stretching is found only in the literature of biological samples [75]

**Table 2:** Major Raman spectrum changes from ABS pure identified.

Wavenumber (cm^−1^)	Raman Spectrum Changes
618	A small shift of Raman line at 616—Linear drop in signal as the concentration of Si_3_N_4_ increases
1000	A small shift of Raman line at 997—Linear drop in signal as the concentration of Si_3_N_4_ increases
1029	A small shift of Raman line at 1027—Linear drop in signal as the concentration of Si_3_N_4_ increases
1578	Linear drop in signal as the concentration of Si_3_N_4_ increases
1600	Linear drop in signal as the concentration of Si_3_N_4_ increases
2234	Linear drop in signal as the concentration of Si_3_N_4_ increases
2908	A broad decrease in the range between 2875–2960 cm^−1^
3057	A broad decrease in the range between 3040–3073 cm^−1^

## Data Availability

The data presented in this study are available upon request from the corresponding author.

## Supplementary Materials

The following supporting information can be downloaded at: https://www.mdpi.com/article/10.3390/nano13101588/s1, Figure S1: 3D printing settings and mechanical test samples geometry, following the corresponding international standard of testing.
