# Supplementary material for: Optimizing the Rheological and Thermomechanical Response of Acrylonitrile Butadiene Styrene/Silicon Nitride Nanocomposites in Material Extrusion Additive Manufacturing
Source: Nanomaterials (Basel). 2023 May 9;13(10):1588. doi: 10.3390/nano13101588 (PMC10221879; doi:10.3390/nano13101588)
Supplement: Supplementary file 1 [file nanomaterials-13-01588-s001.zip › nanomaterials-2385545-supplementary.pdf]

Parameters	Value	Units
ABS Si_3N_4 content	0.0–2.0–4.0–6.0–8.0–10.0	%
Printing orientation	±45	deg
Nozzle Temperature	270	°C
Bed Temperature	80	°C
Layer thickness	0.2	mm
Number of perimeters	2	–
Fill Density	100	%
Travel speed	40	mm/sec

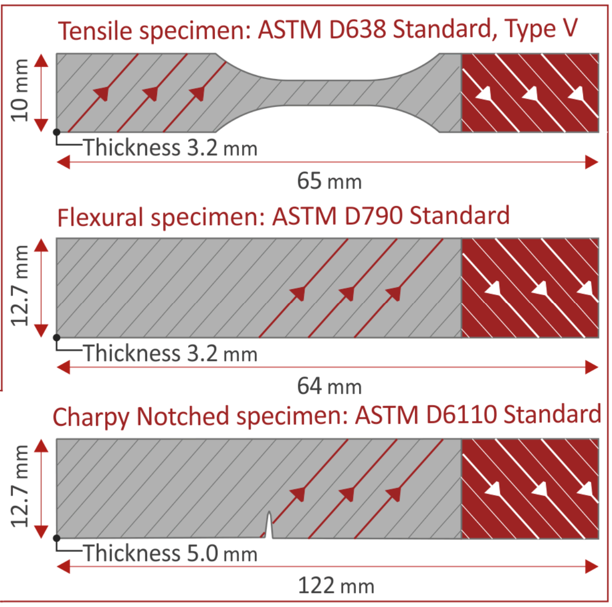

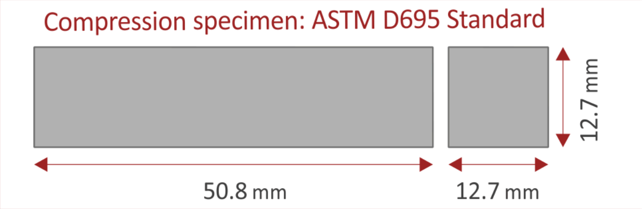

Figure S1. 3D printing settings and mechanical test samples geometry, following the corresponding international standard of testing.
